# Balancing glycolytic flux: the role of 6-phosphofructo-2-kinase/fructose 2,6-bisphosphatases in cancer metabolism

**DOI:** 10.1186/2049-3002-1-8

**Published:** 2013-02-04

**Authors:** Susana Ros, Almut Schulze

**Affiliations:** 1 Gene Expression Analysis Laboratory, Cancer Research UK London Research Institute, 44 Lincoln’s Inn Fields, London, WC2A 3LY, UK

**Keywords:** Cancer metabolism, Fructose 2,6-bisphosphate, PFK-2/FBPase-2

## Abstract

The increased glucose metabolism in cancer cells is required to fulfill their high energetic and biosynthetic demands. Changes in the metabolic activity of cancer cells are caused by the activation of oncogenes or loss of tumor suppressors. They can also be part of the metabolic adaptations to the conditions imposed by the tumor microenvironment, such as the hypoxia response. Among the metabolic enzymes that are modulated by these factors are the 6-phosphofructo-2-kinase/fructose 2,6-bisphosphatases (PFKFBs), a family of bifunctional enzymes that control the levels of fructose 2,6-bisphosphate (Fru-2,6-P_2_). This metabolite is important for the dynamic regulation of glycolytic flux by allosterically activating the rate-limiting enzyme of glycolysis phosphofructokinase-1 (PFK-1). Therapeutic strategies designed to alter the levels of this metabolite are likely to interfere with the metabolic balance of cancer cells, and could lead to a reduction in cancer cell proliferation, invasiveness and survival. This article will review our current understanding of the role of PFKFB proteins in the control of cancer metabolism and discuss the emerging interest in these enzymes as potential targets for the development of antineoplastic agents.

## Review

Cancer cells alter their metabolism to support continuous cell growth and proliferation in a challenging environment. Many cancer cells display high rates of aerobic glycolysis, a phenomenon historically known as the Warburg effect [1]. Oncogenes such as *RAS*, *SRC* and *MYC* have been found to enhance glycolysis by increasing the expression of glucose transporters and glycolytic enzymes [2,3]. Moreover, hypoxia-inducible factor (HIF), a key transcription factor that regulates the adaptation of cells to hypoxic conditions and is frequently deregulated in cancer, also induces the expression of genes involved in glycolysis [4]. It has therefore been concluded that genetic alterations that cause tumorigenesis are also responsible for the regulation of glycolysis in cancer cells (reviewed in [5]).

**Figure 1:**
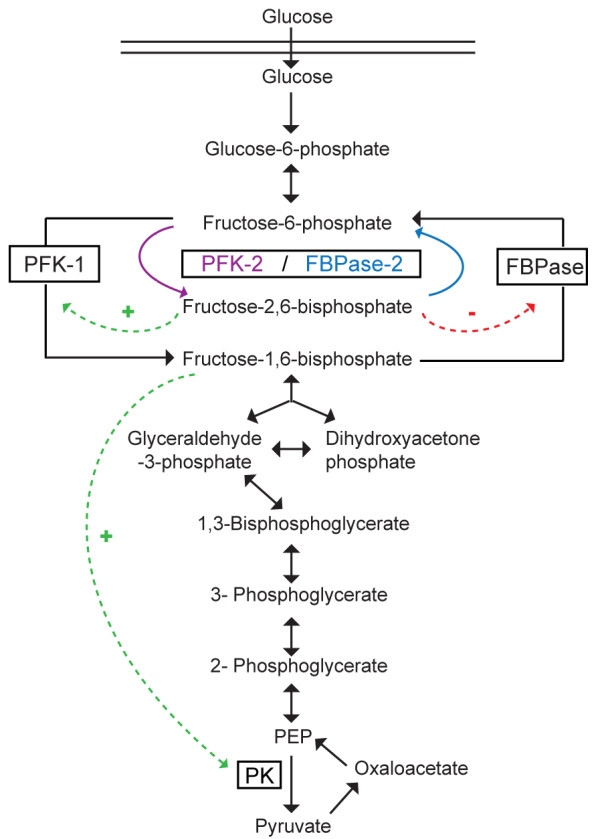
**PFK-2/FBPase-2 control of glycolysis and gluconeogenic pathways.** Overview of glycolysis and gluconeogenesis. Enzymes: phosphofructokinase (PFK-1), fructose 1,6-bisphosphatase (FBPase), 6-phosphofructo-2-kinase/fructose 2,6-bisphosphatases (PFK-2/FBPase-2), pyruvate kinase (PK). Fructose 2,6-bisphosphate is an activator of PFK-1 and inhibitor of FBPase. Fructose 1,6-bisphosphate is an activator of PK. The rest of the enzymes and cofactors have been omitted for simplicity.

Among the glycolytic enzymes that are induced in cancer are the 6-phosphofructo-2-kinase/fructose 2,6-bisphosphatases (PFK-2/FBPase-2), a family of bifunctional enzymes that control the levels of fructose 2,6-bisphosphate (Fru-2,6-P_2_). These enzymes catalyze the synthesis of Fru-2,6-P_2_ from fructose 6-phosphate (Fru-6-P) and ATP, a reaction that occurs at the N-terminal 6-phosphofructo-2-kinase domain (Figure 1). Conversely, PFK-2/FBPase-2 also catalyzes the reverse reaction, the hydrolysis of Fru-2,6-P_2_ to fructose 6-phosphate (Fru-6P) and inorganic orthophosphate at the C-terminal fructose 2,6-bisphosphatase domain (Figure 1). Both catalytic domains are present in the same polypeptide that functions within a homodimeric protein complex [6,7].

Fru-2,6-P_2_ is a powerful allosteric activator of phosphofructokinase 1 (PFK-1), the enzyme that controls one of the most critical steps of glycolysis [8-11]. The tetrameric enzyme PFK-1 catalyzes the conversion of Fru-6-P and ATP to fructose 1,6-bisphosphate and ADP (Figure 1). Interestingly, PFK-1 activity is inhibited by ATP, citrate or fatty acids, thereby adjusting glycolytic activity to environmental conditions and cellular metabolic demands. Indeed, inhibition of PFK-1 by ATP is part of the negative feedback loop that limits glycolytic flux under aerobic conditions (Pasteur effect) and allosteric activation of PFK-1 by Fru-2,6-P_2_ relieves this inhibition [12]. Increased levels of Fru-2,6-P_2_ would therefore allow transformed cells to maintain a high glycolytic flux despite the presence of ATP. However, unlike PFK-1, PFK-2 is not affected by ATP concentrations. Interestingly, inorganic orthophosphate stimulates PFK-2, while phosphoenolpyruvate and citrate can inhibit it. PFK-2 activity is also inhibited by sn-glycerol 3-phosphate, which is competing with Fru-6-P for binding to the catalytic site [13]. sn-glycerol 3-phosphate also stimulates the FBPase-2 activity, and is capable of partially reversing the inhibition of the enzyme by Fru-6-P [13]. GTP also stimulates the FBPase-2 activity [14].

Fru-2,6-P_2_ not only controls the PFK-1 reaction but also controls the reverse reaction in the gluconeogenic pathway by inhibiting fructose 1,6-bisphosphatase (FBPase) [8].

It is clear that by modulating the levels of Fru-2,6-P_2_, PFK-2/FBPase-2 enzymes could be crucial players in the regulation of the metabolic activity of cancer cells.

### The *PFKFB* genes

**Figure 2:**
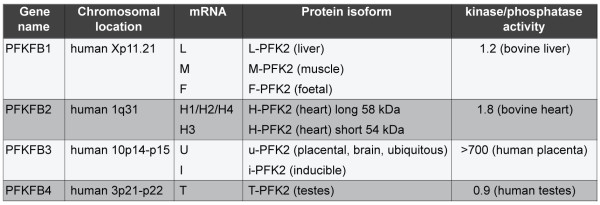
**Relative activity of 6-phosphofructo-2-kinase (PFK-2)/fructose 1,6-bisphosphatase (FBPase-2).** Properties of the PFK-2/FBPase-2 enzymes. Information on chromosomal location and relative enzymatic activity of the different isoforms has been taken from Okar *et al. Trends BiochemSci* (2001) [7].

There are several PFK-2/FBPase-2 isoenzymes in mammals, which are encoded by four different genes, *PFKFB1* to *PFKFB4*, located on different chromosomes (Figure 2). Their genomic organization reveals how each gene codes for several isoforms by differential splicing, and for even more mRNAs due to the presence of several promoters and 5’ non-coding exons. Despite the high sequence homology within their core catalytic domains, different PFK-2/FBPase-2 isoenzymes display distinct regulatory and kinetic properties. Importantly, it has been shown that cells can coexpress several PFK-2/FBPase-2 isoforms suggesting that different isoenzymes cooperate in the regulation of Fru-2,6-P_2_ levels [15,16].

### *PFKFB1*

The *PFKFB1* gene contains 17 exons and encodes 3 different mRNAs (L, M and F) that are derived from different promoters and differ only within their first exon [17,18]. The first exon of the L isoform (exon 1_L_, L-PFK2) codes for 32 amino acids and gives rise to a protein that carries a serine residue at position 32, which can be targeted by phosphorylation (discussed in detail below). This isoform is expressed in liver, skeletal muscle and white adipose tissue. The first exon of the M isoform (exon 1_M_, M-PFK2) only codes for nine amino acids, none of which provides a substrate for phosphorylation. The M promoter targets expression of this isoform to skeletal muscle and white adipose tissue. Promoter F generates the F isoform (non-coding 1_Fa_ and 1_Fb_ and part of 1_M_ exon_,_ F-PFK2) that is expressed in fibroblasts and fetal tissue.

### *PFKFB2*

The *PFKFB2* gene encodes the heart isoenzyme (H-PFK2). The *PFKFB2* gene encodes four different mRNAs that are derived from different promoters and that vary only in non-coding sequences at the 5’ end [19,20]. The resulting proteins thus differ in their C-terminal amino acid sequence [20]. Alternative splicing of the terminal exon 15 results in its two main isoforms (58 and 54 kDa). However, it is not known how these different 5’ ends relate to the three mRNAs (H1, H2 and H4) that encode the 58-kDa isoform or the H3 mRNA that encodes the 54-kDa isoform.

### *PFKFB3*

The *PFKFB3* gene codes for an isoenzyme originally cloned from bovine brain [21] and from human placenta [22]. It contains at least 19 exons and has been shown to generate at least 6 different transcripts by alternative splicing. The resulting polypeptides differ in the length of their C-terminal variable regions that are encoded by the last seven exons. Alternative splicing of exon 15 generates the two main isoforms that differ by a short C-terminal sequence: the ubiquitous PFK2 (u-PFK2, 15 exons) and the inducible PFK2 (i-PFK2, 16 exons) isoforms [23]. Four additional splice variants have also been reported in human brain [24].

### *PFKFB4*

The *PFKFB4* gene encodes the testes isoenzyme (T-PFK2), since it was originally identified in testes [25]. The *PFKFB4* gene contains at least 14 exons, and several splice variants of the *PFKFB4* mRNA have been identified not only in testes but also in different rat tissues [26]. These include transcripts carrying deletions or inserts within the FBPase-2 region as well as some with varying length and amino acid sequence of the C-terminal part. However, the PFK-2 catalytic domains are identical in all variants. This could indicate a possible role of different *PFKFB4* splice variants in cell-specific and/or tissue-specific regulation of glycolysis and requires further investigation.

### TIGAR: a PFK-2/FBPase-2-like enzyme

TIGAR (C12orf5), for ‘TP53-Inducible Glycolysis and Apoptosis Regulator’, shows functional similarities to the bisphosphatase activity of PFK-2/FBPase-2 s [27]. Although the overall sequence similarity between TIGAR and FBPase-2 is relatively weak, the regions in the FBPase-2 domain that have previously been identified as essential for catalytic activity are well conserved in TIGAR.

### Differential PFK-2/FBPase-2 activities

**Figure 3:**
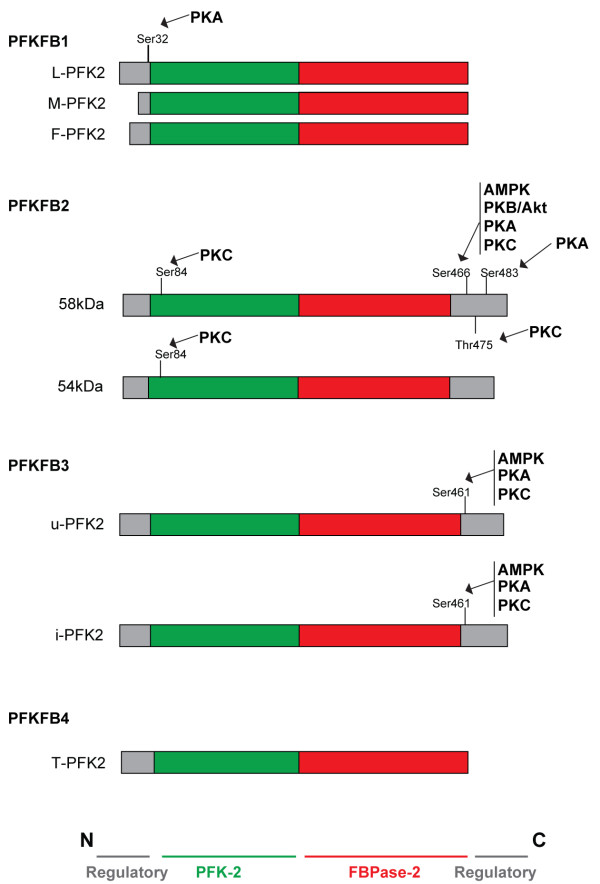
**Domain organizations and phosphorylation of 6-phosphofructo-2-kinase (PFK-2)/fructose 1,6-bisphosphatase (FBPase-2) isoenzymes.** The N-terminal PFK-2 domain is shown in red, the C-terminal FBPase-2 domain is shown in blue and the regulatory domains are shown in green. Phosphorylation sites and the kinases responsible of their phosphorylation are indicated on each main isoforms. The domain structure of human PFK-2/FBPase-2 enzymes is based on Rider et al. Biochem J (2014) [10].

The structural organization of the different PFK-2/FBPase-2 isoenzymes is highly similar, with the kinase and bisphosphatase domains in the center flanked by regulatory regions (Figure 3). The sequence of the catalytic core is highly conserved while the N-terminal and C-terminal extremes of the proteins show more divergence, being where the regions that modulate the kinase and bisphosphatase activities are located. The differences in kinase and bisphosphatase activities between the different isoenzymes are presumably caused by structural variations within these regulatory regions.

6-Phosphofructo-2-kinase/fructose 2,6-biphosphatase 1 (PFKFB1) and PFKFB2 seem to have similar relative kinase and bisphosphatase activities (Figure 2) [7]. In contrast, PFKFB3 has a substantially higher kinase activity and strongly favors the formation of Fru-2,6-P_2_, resulting in an enhanced glycolytic rate [7]. The activities of PFKFB4 are again more balanced, with the bisphosphatase activity being slightly higher [7]. However, since the different PFK-2/FBPase-2 enzymes are also regulated by post-translational modifications (discussed below), their relative kinase and bisphosphatase activity could vary according to the cellular context.

### Post-translational modifications of PFK-2/FBPase-2 enzymes

Several PFK-2/FBPase-2 isoforms are subject to post-translational modifications that modulate the relative activities of their catalytic domains. This places the production of Fru-2,6-P_2_ under the control of cellular signaling pathways and facilitates the regulation of glycolytic activity in response to extracellular stimuli such as hormones or growth factors. Therefore, Fru-2,6-P_2_ plays an important role in the adaptation of organisms, tissues within them and to changes in their environmental or metabolic state.

The liver, muscle and fetal isoforms of PFKFB1 (L-PFK2, M-PFK2 and F-PFK2 respectively) are transcribed from the same gene, but only L-PFK2 contains a serine residue in position 32 of its C-terminal regulatory domain [6] (Figure 3). This is consistent with its specific physiological role as liver cells need to modulate Fru-2,6-P_2_ levels to facilitate the production of glucose to fulfill the metabolic demand of other tissues. In response to glucagon, cyclic AMP-dependent protein kinase (PKA) phosphorylates Ser32 in the liver isoform of PFKFB1, leading to inactivation of its PFK-2 activity while activating its FBPase-2 function. This decreases glycolytic flux while increasing gluconeogenesis in liver cells [28].

While phosphorylation of L-PFK2 results in a decrease in its kinase activity, phosphorylation of the cardiac isoform, known as H-PFK2 (PFKFB2), results in an increase in this activity. Insulin stimulates glycolysis in the heart by increasing glucose transport and activating H-PFK2. Two serine residues, Ser466 and Ser483, within the C-terminal regulatory domain of PFKFB2 can be phosphorylated by protein kinase B (Akt/PKB) in response to insulin [29] (Figure 3). Thus, expression of an insulin-dependent PFK2 in the heart could be involved in directing the increased levels of glucose-6-phosphate, that result from the insulin-stimulated glucose transport, towards the synthesis of pyruvate [30]. Adrenalin promotes phosphorylation of the same sites by PKA and also stimulates glycolysis [31]. However, the physiological significance of phosphorylation of Ser84, Ser466 and Thr475 of H-PFK2 by protein kinase C (PKC) is not completely understood. It seems that phosphorylation of Ser466 or Thr475 does not change the enzyme activity. This might be due to the fact that the phosphorylation at Ser84 possibly counteracts the effects of phosphorylation at the activating C-terminal sites [31,32]. Activation of the AMP-activated protein kinase (AMPK) during ischemia leads to phosphorylation of H-PFK2 Ser466 [33], which increases the levels of Fru-2,6-P2 and stimulates glycolysis. It has also been reported that phosphorylation of Ser466 and Ser483 in the 58-kDa isoform of H-PFK2 (PFKFB2) increases its affinity to Fru-6-P, whereas dephosphorylation of the same residues promotes its FBPase-2 activity [34]. Interestingly, these phosphorylation sites are absent from the 54-kDa isoform indicating that this isoform may have an overall reduced kinase activity and could be less responsive to cellular signaling processes (Figure 3). It is also possible that the two splice variants have opposite functions in the regulation of glycolysis.

Both the ubiquitous (u-PFK2) and inducible (i-PFK2) isoforms of PFKFB3 can be phosphorylated by AMPK at Ser461 within their C-terminal regulatory domain. Phosphorylation by AMPK activates the PFK-2 activity. This site is equivalent to Ser466 in the 58-kDa isoform of H-PFK2 [33,35], which is phosphorylated in response to insulin. It would be interesting to investigate whether the same occurs in PFKFB3. Ser461 can also be phosphorylated by PKC and PKA [36] making it responsive to multiple external signals. As this region is sensitive to alternative splicing, the structural configuration around this site may vary between different splice variants. Although the functional consequences of having different C-terminal splice variants of PFKFB3 are unknown, an examination of their activity would be useful to further understand the regulation of this isoenzyme. Interestingly, the activity of PFKFB3 is also regulated by modulation of protein stability. PFKFB3, but not the other isoenzymes, contains a KEN box that is recognized by the anaphase-promoting complex/cyclosome (APC/C-cdh1), an E3 ubiquitin ligase complex that plays an essential role in G1 phase and mitosis through the degradation of several cell cycle proteins [37]. The regulatory component of this complex, Cdh1, has been shown to be downregulated during malignant progression in a murine B lymphoma cell line [38] and mice heterozygous for *cdh1* are more susceptible to spontaneous tumor formation [39]. This decrease in APC/C-Cdh1 activity leads to the accumulation of PFKFB3 and enhances the glycolytic flux in malignant cells [37], demonstrating that proliferation and the induction of aerobic glycolysis are both essential components of neoplastic transformation.

To date, no post-translational modifications have been described for PFKFB4, making this isoform potentially less dynamic. However, it is likely that PFKFB4 activity is modulated in response to changes in the concentration of substrate and product or the presence of activators or inhibitors.

### PFK-2/FBPase-2 enzymes in hypoxia

Hypoxia is an important component of the tumor microenvironment and can regulate several processes important for tumor formation, including tumor angiogenesis and metastasis formation. Hypoxia also regulates the metabolic activity of cancer cells and induces glycolysis. Adaptation to hypoxic conditions is largely mediated by HIF, a transcription factor that regulates a distinct transcriptional program [40]. HIF is activated in hypoxia through the stabilization of its regulatory α subunits and induces the expression of its target genes, including glucose transporters and glycolytic enzymes. Notably, HIF can be active in cancer cells even under non-hypoxic conditions, thereby contributing to the enhanced glycolytic activity observed in cancer cells [41,42].

Interestingly, both *PFKFB3* and *PFKFB4* are known to be components of the HIF-mediated response to hypoxia. *PFKFB3* is a hypoxia-inducible gene that is stimulated through the interaction of HIF-1α with a consensus hypoxia response element (HRE) within its promoter region [43]. In addition, hypoxic induction of PFKFB4 is mediated by a HRE in the promoter region of the *PFKFB4* gene [44]. Other studies have demonstrated that *PFKFB1, PFKFB2, PFKFB3 andPFKFB4* mRNAs are induced in organs that are exposed to hypoxic conditions, although *PFKFB3* presented the highest induction in this study [16].

Overall, the finding that PFK-2/FBPase-2 enzymes are induced in hypoxia suggests their important role in the glycolytic phenotype of cancer cells. In this context they would enable metabolic adaptation to the hypoxic environment promoting cell survival.

### PFK-2/FBPase-2 enzymes in cancer

**Figure 4:**
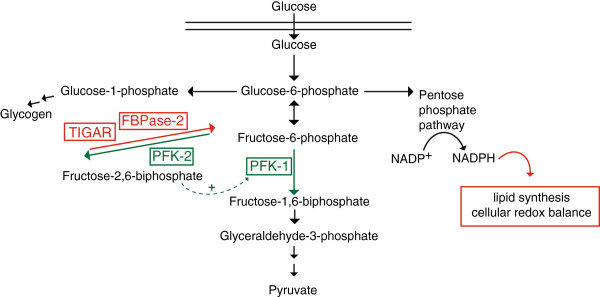
**Role of 6-phosphofructo-2-kinase (PFK-2)/fructose 1,6-bisphosphatase (FBPase)-2 in the balance between glycolytic flux and redox regulation.** Glucose-6-phosphate fulfills glycolysis, glycogen synthesis and pentose phosphate pathways. The scheme shows how the PFK-2 or the FBPase-2 activity could be crucial in controlling the balance between glycolytic flux and redox regulation by the pentose phosphate pathway for nicotinamide adenine dinucleotide phosphate (NADPH) production. Enzymes: phosphofructokinase (PFK-1), phosphofructokinase 2/fructose 2,6-bisphosphatase (PFK-2/FBPase-2), TIGAR (FBPase-2 activity). Fructose 2,6-bisphosphate is an activator of PFK-1 and inhibitor of FBPase.

Several studies have demonstrated that some cancer cell lines produce markedly elevated levels of Fru-2,6-P_2_ when compared to non-malignant cells [45-47]. One of the important functions of enhanced glucose uptake and glycolysis in cancer cells is to generate metabolic intermediates that are important for cell growth and survival (Figure 4). Metabolism of glucose through the oxidative or non-oxidative arms of the pentose phosphate pathway (PPP) generates ribose-5-phosphate, a key intermediate in nucleotide biosynthesis. The oxidative arm of the PPP also generates nicotinamide adenine dinucleotide phosphate (NADPH), which is required for nucleotide and fatty acid biosynthesis. Moreover, NADPH is also important for the maintenance of cellular redox balance. The enhanced metabolic activity of cancer cells can result in increased levels of reactive oxygen species (ROS) and to oxidative damage. ROS-induced-damage can be repaired due to NADPH providing the reducing power for the glutathione (GSH) and thioredoxin (TRX) systems [48]. In this regard, it is hypothesized that the fructose 2,6-bisphosphatase activities of the PFK-2/FBPase-2 enzymes could also allow accumulation of glycolytic intermediates for biosynthetic processes and redox control (Figure 4).

As the increased levels of Fru-2,6-P_2_ could allow transformed cells to maintain a high glycolytic flux, several studies have investigated the potential roles of the different PFK-2/FBPase-2 enzymes in cancer metabolism. All PFKFB mRNAs have been reported to be overexpressed in the human lung cancers when compared with corresponding normal tissues [49].

However, there has not been a great deal of characterization of the possible role of PFKFB1 in cancer to date. Expression of the fetal isoform of PFKFB1 has been shown to be induced in proliferating cells resulting in an increased glycolytic rate [18].

PFKFB2 has been shown to be upregulated as a consequence of the transcriptional changes orchestrated by the androgen receptor in prostate cancer cells, with possible control through the AR-CAMKII-AMPK signaling pathway [50]. Moreover, expression of PFKFB2 is induced in LNCaP prostate cancer cells after androgen treatment by the direct recruitment of the ligand-activated androgen receptor to the *PFKFB2* promoter [51]. Depletion of *PFKFB2* expression resulted in a reduced glucose uptake and lipogenesis, suggesting that the induction of *de novo* lipid synthesis by androgen requires the transcriptional upregulation of *PFKFB2* in prostate cancer cells [51].

Due to its increased kinase activity, PFKFB3 has been suggested as the isoenzyme most likely to contribute to the high glycolytic activity observed in transformed cells [52]. The inducible isoform of PFKFB3 (i-PFK2) has been reported to be overexpressed in several human cancer cell lines if compared to normal cells, and has been shown to be required for tumor cell growth *in vitro* and *in vivo*[53]. PFKFB3 mRNA is induced in hypoxia though a HIF-1α dependent mechanism in cancer cell lines from different tissues including glioblastoma, gastric and pancreatic cancer [54]. In contrast, the *PFKFB3* gene, in particular the i-PFK2 splice variant, has been recently shown to be a target for loss of heterozygosity on 10p14-p15, which is common in high-grade gliomas [55]. A reduction of total PFKFB3 level by allelic deletion might lead to decreased Fru-2,6-P_2_ levels, which might result in decreased rate of glycolysis: a hallmark of glioblastomas [56,57]. However, other types of cancers, such as breast, colon, lung, pancreatic, prostatic and ovarian, express high levels of PFKFB3 protein relative to the non-malignant adjacent tissue [58]. Furthermore, PFKFB3 protein is highly phosphorylated at Ser461, the consensus site for AMPK, PKA and PKC, in colon and breast carcinoma compared to epithelial cells from normal tissue, suggesting enhanced PFK-2 activity in cancer [35]. It has been shown recently that elevated expression of the tumor suppressor phosphatase and tensin homolog (PTEN) negatively impacts on glycolysis by promoting APC/C-Cdh1 activity resulting in the downregulation of the PFKFB3 protein [59]. Given that PTEN is frequently mutated or deleted in human cancer, it would be interesting to further investigate the correlation between loss of PTEN and a possible increase in PFKFB3 protein, which could then impact on glycolytic activity. Moreover, silencing of *PFKFB3* has been reported to decrease Fru-2,6-P_2_ levels and glycolytic rate in HeLa cells, thereby reducing cell viability and anchorage-independent growth [60]. Interestingly, introduction of oncogenic *H-ras*^*V12*^ promotes anchorage-independent growth of primary lung fibroblasts derived from *PFKFB3*^+/+^ but not from *PFKFB3*^+/−^ mice [15]. *PFKFB3*^+/−^ fibroblasts have reduced levels of Fru-2,6-P_2_ compared to wild-type cells. This might expose PFK-1 to the inhibitory effects of ATP thereby abolishing the capacity of oncogenic *RAS* to confer the capacity for anchorage-independent growth, suggesting that PFKFB3 may be a highly selective target for the development of antineoplastic agents.

*PFKFB4* expression is induced in breast, colon, lung, gastric and pancreatic cancer cell lines [49,54,61,62]. Interestingly, it has been recently demonstrated that PFKFB4 plays an essential role in the survival of glioma stem-like cells [63]. Knockdown of *PFKFB4* resulted in downregulation of lactate secretion and ATP production and ultimately induced apoptosis in these cells. This study also demonstrated that brain cancer stem-like cells have increased *PFKFB4* mRNA expression when compared to that of adult normal brain tissue, while the mRNA of *PFKFB3* is downregulated. These findings suggested that *PFKFB4* is the main PFK-2/FBPase-2 isoenzyme that regulates glycolytic flux in malignant glioma cells. Interestingly, PFKFB4 silencing has also been demonstrated to be detrimental for prostate cancer cell survival [64]. Silencing of *PFKFB4* resulted in increased levels of Fru-2,6-P_2_ in prostate cancer cells. This led to the conclusion that in response to *PFKFB4* depletion, metabolic intermediates are diverted towards glycolysis and away from the oxidative arm of the PPP, resulting in reduced levels of NADPH production. Prostate cancer cells may then selectively rely on PFKFB4 for managing ROS accumulation, since treatment with a ROS scavenger rescued the viability defect in the PFKFB4-knockdown prostate cancer cells. PFKFB4 mRNA expression was also found to be higher in metastatic prostate cancer when compared with primary tumors. Notably, PFKFB4 was not only required for the survival of prostate cancer cells, but also for other cancer cell lines from different tissues. While the role of this isoenzyme in different cancer types needs further investigation, these results suggest that it could be a potential target for cancer therapy.

The potentially opposing effects of the different PFK-2/FBPase-2 in regulating Fru-2,6-P_2_ levels in some cancer cells may seem contradictory. Further investigation in the relationship between Fru-2,6-P_2_ concentration and which isoforms are expressed in these tumors would be helpful to understand this paradigm. However, it is likely that cancer cells express different levels of the different PFK-2/FBPase-2 enzymes and even modulate the relative kinase and bisphosphatase activity according to their metabolic needs in a spatial and/or temporal manner. Cancer cells may experience conditions of intermittent hypoxia due to insufficient vascularization of some tumor areas. Under these conditions, induction of glycolysis may be required to support anaerobic ATP production for which the kinase activity of PFK-2/FBPase-2 enzymes would be essential. However, the high biosynthetic demand of rapidly proliferating tumor cells also requires the production of large amounts of NADPH and the bisphosphatase activity may be indispensable to limit glycolytic activity and divert metabolites for NADPH production. It is therefore possible that cancer cells regulate the activity of different PFK-2/FBPase-2 enzymes in a tight temporal and spatial manner to meet their metabolic demands and therefore maintain viability.

TIGAR could also play an important role in this complexity and participate in shaping the metabolic profile of the cell. It has been demonstrated that TIGAR works as an FBPase-2 and diverts glucose intermediates from glycolysis into the pentose phosphate pathway [27]. Then TIGAR promotes the production of NADPH to allow the generation of reduced glutathione to protect against ROS, and facilitates the formation of ribose 5-phosphate for nucleotide synthesis. It was also reported that overexpression of TIGAR in cancer cells led to ROS quenching and protection from p53-mediated apoptosis as a result of genotoxic stress and DNA damage [27]. Therefore, the reduction of glycolytic flux through the induction of TIGAR is consistent with the function of p53 as a tumor suppressor. Interestingly, inhibition of the pentose phosphate pathway can also lead to the activation of p53, suggesting the presence of a feedback loop that can restore pentose phosphate activity through induction of p53 and TIGAR [65]. Notably, p53 was also shown to reduce the production of NADPH by binding and inhibiting glucose-6-phosphate dehydrogenase (G6PD), the first and rate-limiting enzyme of the pentose phosphate pathway [66]. It is believed that the increased G6PD activity may be dominant over the loss of TIGAR induction in p53 mutant/loss cancer cells. Furthermore, TIGAR was found to be expressed in several cancer cell lines in a p53-independent manner and therefore may have additional roles in the survival and proliferation of transformed cells [27].

It is clear that PFK-2/FBPase-2 enzymes and TIGAR are important players in the control of cancer cell metabolism. However, it should also be considered that PFK-2/FBPase-2 enzymes might have other functions in cancer cells that are unrelated to their role as glycolytic regulators. In this context, a recent study showed that the C-terminal domain of PFKFB3 variant 5 localizes the enzyme to the nucleus where Fru-2,6-BP increases the expression and activity of cyclin-dependent kinase-1 promoting cell proliferation [52].

### Targeting PFK-2/FBPase-2 enzymes for cancer therapy

As outlined above, PFK-2/FBPase-2 enzymes play an important role in tumor metabolism and cancer cell survival. Given this, it is not surprising that they have been considered as potential targets for cancer therapy. These efforts have thus far mainly been concentrated on the inhibition of the PFK-2 activity of PFKFB3, as it is induced by several oncogenes as well as hypoxia and may contribute to the high glycolytic activity observed in cancer cells. Several compounds have been developed to date. One of these compounds, *N*-bromoacetiletanolamine phosphate, was originally developed as a synthetic substrate analogue for fructose bisphosphate aldolase, the glycolytic enzyme that catalyzes the reversible conversion of fructose 1,6-bisphosphate into dihydroxyacetone phosphate and glyceraldehyde 3-phosphate (Figure 1). However, it was subsequently shown to be an irreversible inhibitor of PFK-2 in several cancer cell lines [67]. Clem *et al.* identified a small molecule inhibitor of PFK-2 named 3-(3-pyridinyl)-1-(4-pyridinyl)-2-propen-1-one (3-PO) [68]. This compound has been shown to decrease glucose uptake and Fru-2,6-P_2_ levels *in vivo,* and to suppress tumorigenic growth of breast, leukemia and lung adenocarcinoma cells. However, due to the strong homology of all four PFKFB proteins in their PFK-2 domain, it is possible that 3-PO not only inhibits PFKFB3 but also the other isoenzymes. Two additional PFKFB3 inhibitors have been recently developed using a structure-based approach: N4A and its derivative YN1 [69]. These compounds should have higher specificity as they are targeted towards the fructose 6-phosphate-binding site rather than the ATP-binding pocket. Since the activity of these compounds towards other PFKFB proteins has not yet been established, their antiproliferative effect on cancer cells may not be solely ascribed to the inhibition of PFKFB3. Consequently, it may be necessary to develop inhibitors that are highly selective towards PFKFB3 to avoid toxicity due to the inhibition of other PFK-2/FBPase-2 enzymes, particularly for tissues such as the liver that support metabolic homeostasis at an organismal level.

Strategies to inhibit PFK-2 activity, in particular for PFKFB3, may be successful in reducing the high glycolytic activity of cancer cells. However, recent evidence suggests that the FBPase-2 activity, specifically that of PFKFB4, may also be important for cancer cell survival [64]. Knockdown of PFKFB4 blocked prostate cancer cell growth and remarkably induced regression of prostate tumor xenografts, confirming that prostate cancer cells are dependent on PFKFB4 for survival. Moreover, the requirement of PFKFB4 for cell survival seems to be extended beyond prostate cancer because other cancer cell lines were also sensitive to PFKFB4 knockdown. Together, these findings implicate PFKFB4 as a potential therapeutic target. However, no strategies for the selective inhibition of the FBPase-2 activity have been developed to date. A major obstacle for this approach is the general lack of unique topological features within phosphatase domains, which limits the specificity of binding of candidate compounds. Despite these challenges, FBPase-2 activity may still emerge as an important target for cancer therapy. Enhancing glycolytic flux while depleting metabolites from the pentose phosphate pathway should cause irreversible cellular damage due to increased ROS accumulation. It will therefore be interesting to evaluate whether systemic inhibition of the FBPase-2 activity of PFKFB4 will provide antitumor efficacy in different types of cancer.

## Conclusions

We are gaining significant knowledge about the function and regulation of PFK-2/FBPase-2 enzymes in cancer cells. Allosteric regulation of glycolysis by Fru-2,6-P_2_ allows cancer cells to precisely balance their glycolytic flux to fulfill their bioenergetics and biosynthetic demands. Disrupting this balance is likely to limit the capacity of cancer cells to adapt to the metabolic constraints imposed by the tumor microenvironment. However, further research into their expression, their relative activities, mechanisms of allosteric regulation, localization and post-translational modification is required to fully understand the role of these enzymes in cancer metabolism. Moreover, another level of complexity could be added and functions unrelated to glycolysis could be attributed to these enzymes in cancer. Despite these open questions, the evidence accumulated to date suggests that PFK-2/FBPase-2 enzymes could be attractive targets for cancer treatment.

## Competing interests

The authors declare that AS provides minor consultancy work for Astra Zeneca. The authors declare no competing interests.

## Authors’ contributions

SR and AS wrote the article. Both authors read and approved the final manuscript.

## Authors’ information

SR: postdoctoral fellow at Gene Expression Analysis Laboratory, Cancer Research UK London Research Institute. AS: Cancer Research UK London Research Institute.
